# X‐Linked USP11 Drives Depression‐Like Behaviors by Stabilizing CK2α and Disrupting Mitochondrial Function

**DOI:** 10.1002/cns.70934

**Published:** 2026-06-02

**Authors:** Yuqi Feng, Ningyuan Li, Lingfeng Zhang, Wei Wang, Hao Duan, Siqi Sun, Ruiling Li, Yuhui Zhang, Xinhua Song, Yingyue Zhang, Honghan Zhang, Zhaowen Nie, Hanchun Yan, Chao Wang, Zhongchun Liu

**Affiliations:** ^1^ Department of Psychiatry Renmin Hospital of Wuhan University Wuhan China; ^2^ Clinical College of Traditional Chinese Medicine Hubei University of Chinese Medicine Wuhan China; ^3^ Taikang Center for Life and Medical Sciences Wuhan University Wuhan China; ^4^ State Key Laboratory of Metabolism and Regulation in Complex Organisms Wuhan University Wuhan China

**Keywords:** casein kinase II subunit alpha (CK2α), depression, mitochondrial dysfunction, mitochondrial fusion and fission, ubiquitination, ubiquitin‐specific peptidase 11 (USP11)

## Abstract

**Aims:**

To explore the role and mechanism of X‐linked USP11 in mitochondrial dysfunction associated with depression.

**Methods:**

USP11 knockout mice and USP11 overexpression mice in the prefrontal cortex were constructed, and the role of USP11 in mitochondrial dysfunction in depression was evaluated by behavioral tests and quantitative analysis of mitochondrial function changes in the prefrontal cortex. The interaction protein CK2α of USP11 in depression was identified by IP‐MS, and the role of CK2α was confirmed by using the selective inhibitor CX4945. The direct effects of USP11 and CK2α on mitochondrial function were further verified by using primary neurons.

**Results:**

USP11 mediates depression‐like behaviors and impairs mitochondrial function in mice. Mechanistically, USP11 binds to and deubiquitinates CK2α, stabilizing its protein level and promoting mitochondrial dysfunction. The selective inhibitor CX4945 reverses the impairment of neuronal mitochondrial function by CK2α.

**Conclusion:**

This study demonstrates that USP11 directly binds to and deubiquitinates CK2, affecting mitochondrial function in the mPFC and leading to depressive‐like behaviors in mice.

## Graphical abstract

This study reveals that USP11 mediates depressive‐like behaviors and mitochondrial dysfunction in the prefrontal cortex. Mechanistically, USP11 directly binds to and deubiquitinates CK2α, thereby stabilizing its protein level and promoting mitochondrial impairment. Pharmacological inhibition of CK2α with CX4945 reverses USP11‐induced mitochondrial dysfunction, identifying the USP11‐CK2α axis as a potential therapeutic target for depression.

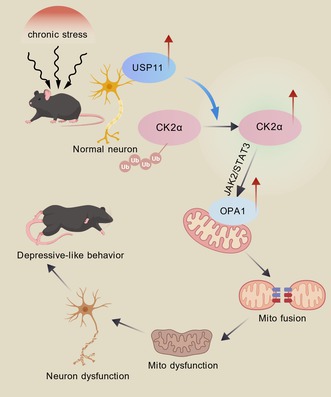

## 1 Introduction

Major depressive disorder (MDD) is a highly heterogeneous psychiatric illness characterized by persistent low mood, cognitive impairment, and an elevated risk of suicide [1, 2], affecting approximately 300 million individuals worldwide (World Health Organization, 2018). Its etiology are multifactorial, involving genetics, psychology, environment, and society, and often accompanied by comorbidities [3, 4, 5]. Mitochondrial dysfunction is presently acknowledged as a contributing factor in major depressive illness. The specific mechanisms encompass mitochondrial DNA mutations, mitochondrial autophagy, mitochondrial dynamics, mitochondrial biogenesis, and mitochondrial energy metabolism [6, 7, 8, 9, 10, 11]. A clinical investigation by Giselli Scaini et al. demonstrated a mitochondrial fusion‐fission imbalance in patients with major depressive disorder [12]. Paola Brivio et al. discovered that mitochondrial fusion is enhanced in the ventral hippocampus of vulnerable rats [13].

USP11 is a deubiquitinating enzyme encoded by the *USP11* gene located on the X chromosome. As a member of the deubiquitinating enzyme family, USP11 serves as a pivotal regulator of ubiquitin‐dependent protein turnover. It modulates the stability and function of target proteins through deubiquitination [14]. USP11 is highly expressed in the human brain and plays a crucial role in various pathological and physiological processes, including cell proliferation, cancer growth and metastasis, chemotherapy resistance, and cerebral hemorrhage [15, 16, 17, 18, 19, 20, 21, 22, 23, 24, 25, 26, 27]. Recent studies have highlighted the significance of USP11 in the context of tau pathology in Alzheimer's disease. Specifically, USP11 can deubiquitinate and promote lysine acetylation of residues 281 and 274 of the tau protein, which facilitates the abnormal aggregation of tau protein during the development of Alzheimer's disease [28]. However, the role and mechanism of USP11 in depression remain unclear.

CK2 (formerly known as casein kinase 2 or CK‐II) is one of the earliest discovered protein kinases, capable of regulating cell proliferation and apoptosis [29, 30, 31, 32, 33]. In mammalian cells, CK2 forms a heterotetramer composed of two catalytic α or α′ subunits and two regulatory β subunits [34, 35]. CK2 has been reported to be involved in various mental disorders. In diseases such as autism, attention deficit hyperactivity disorder, and schizophrenia, the activity of CK2 is crucial for maintaining normal physiological functions and preventing the occurrence of diseases, and it should be protected or even enhanced [36, 37, 38, 39, 40, 41, 42, 43, 44, 45, 46]. In depression, its activity may directly drive the pathological process, so inhibiting CK2 is regarded as a potential therapeutic strategy. Rebholtz showed that the activity of CK2 in the prefrontal cortex is highly correlated with behaviors related to emotions and depression, and its targeted treatment has been proposed for the treatment of depression [47]. Further studies have shown that CK2 can regulate mitochondrial function, exerting its effects through various aspects such as mitochondrial membrane potential [48, 49], mitochondrial autophagy [44, 50], and mitochondrial dynamics [51, 52, 53, 54, 55, 56, 57]. Although CK2 has been studied in various diseases, the specific regulatory mechanism of CK2 in depression remains unclear.

To address this critical knowledge gap, we hypothesized that USP11 mediates the development of depression by stabilizing CK2 through deubiquitination and thereby affecting mitochondrial function. Here, we provide both in vivo and in vitro evidence that USP11 can deubiquitinate CK2, thereby altering its protein abundance and influencing mitochondrial function. This mitochondrial dysfunction leads to depression disorder.

## 2 Materials and Methods

### 2.1 Cell Culture

Human embryonic kidney 293T (HEK293T, CL‐0005, Pricella, Wuhan, China) cells were cultured in Dulbecco's Modified Eagle Medium (DMEM, 15140‐122, Gibco, Thermo Fisher Scientific, Waltham, MA, USA) supplemented with 10% fetal bovine serum (086‐150, Wisent, Saint‐Jean‐Baptiste, QC, Canada) and 1% penicillin–streptomycin (15140–122, Gibco, Thermo Fisher Scientific, Waltham, MA, USA). Human neuroblastoma cells (SK‐N‐SH, CL‐0214, Pricella, Wuhan, China) are cultured in SK‐N‐SH Cell Complete Medium (CM‐0214, Pricella, Wuhan, China). The cells were incubated at 37°C in a humidified environment with 5% CO_2_.

### 2.2 Primary Neuron Culture

Primary neurons were extracted from the embryonic cortex of E16 mice following the established experimental technique. They were inoculated into poly‐L‐lysine‐coated cell culture dishes at a density of 1.0 × 10^6^/cm^2^. The primary neurons were cultured in a neurobasal medium (21103049, Gibco, Thermo Fisher Scientific, Waltham, MA, USA) supplemented with 2% B27 (17504044, Gibco, Thermo Fisher Scientific, Waltham, MA, USA), 1% glutamine (25030081, Gibco, Thermo Fisher Scientific, Waltham, MA, USA), and 1% streptomycin/penicillin (15140‐122, Gibco, Thermo Fisher Scientific, Waltham, MA, USA).

### 2.3 Experimental Animals

Wild type C57BL/6J mice were acquired from Hunan Slack King Laboratory Animal Co. in Changsha, Hunan, China. Usp11 knockout mice on the C57BL/6J genetic background were produced by Cyagen Company (Suzhou, China). The Cas9 protein and two guide RNAs (gRNA‐1: TTTAGTTGTGCAGGATGGCGGGG, gRNA‐2: GGCTACCCATTAAAGCTACATGG) targeting exons 2–9 of the mouse Usp11 gene were co‐injected into fertilized eggs. Embryos were implanted into recipient female mice to produce F0 mice. The genotype of knockout mice was validated using PCR employing two pairs of primers (F1: 5′‐AACACATTAGATGGCTGACAAACAC‐3′, R1: 5′‐TTCCTGAGCCACTTCCTGTTGAC‐3′; F2: 5′‐TCTTATCTCATGCTCACTCTCCC‐3′, R2: 5′‐TTCCTGAGCCACTTCCTGTTGAC‐3′) and subsequent sequencing.

### 2.4 Chronic Unpredictable Mild Stress (CUMS) Model

The experimental design of CUMS remained consistent with previous iterations, with only minor adjustments. The detailed CUMS plan is presented in Table S1.

### 2.5 Behavioral Tests

#### 2.5.1 Open Field Test (OFT)

Mice were situated in the experimental room 24 h before the test. Mice were placed in the center of an open field apparatus (50 × 50 × 35 cm), and their movements were documented for 5 min during the test. Following each mouse trial, 75% ethanol was employed to sanitize the open field apparatus to eliminate any potential odor influence on mouse behavior. The overall distance was assessed using a video‐tracking system (EthoVision XT 11.5, Noldus, Wageningen, Netherlands).

#### 2.5.2 Tail Suspension Test (TST)

Mice were individually suspended by their tails from a vertical bar positioned at the top of a box measuring 30 × 30 × 60 cm. To guarantee stability, sticky medical tape was affixed 2 cm from the tail tip. The duration of immobility was assessed during the final 4 min of a 6‐min testing session. Immobility in the TST was characterized by the lack of any limb or body movements, save those caused by respiration.

#### 2.5.3 Sucrose Preference Test (SPT)

Mice were given two identical water bottles. Initially, both bottles contained water for 24 h, after which they were filled with a 1% sucrose solution for the subsequent 24 h. Water was thereafter eliminated for the following 24 h. One bottle contained water, while the other contained a 1% sucrose solution during the test. The locations of the two bottles were exchanged during the test. The sucrose preference index was determined by the ratio of the volume of 1% sucrose solution ingested by mice to their overall liquid intake.

#### 2.5.4 Forced Swimming Test (FST)

The mice were situated in a transparent glass cylinder with a diameter of 25 cm and a height of 30 cm, filled with water to a depth of 15 cm, maintained at a temperature of 24°C ± 1°C. Each mouse underwent testing for 6 min, with the initial 2 min designated for acclimatization. The duration of the mice's immobility in the water was documented only for the succeeding 4 min.

### 2.6 Transfection

HEK293T cells were introduced into the cell culture dish. Transfection commenced when the cell density attained 50%–70%. Prior to transfection, prepare 1.5 mL of sterile EP tubes, incorporating serum‐free media (03.18001C, Eallbio, Beijing, China) and transfection reagent (TF20121201, Neofect, Waltham, Massachusetts). In a separate EP tube, incorporate serum‐free media and the isolated plasmid DNA, measured according to its concentration. Combine and incubate individually for 5 min; subsequently, add the transfection reagent mixture to the plasmid mixture and incubate for an additional 15 min. Subsequently, incorporate this mixture into the HEK293T cell culture dish. The transfection duration was 6–8 h, after which the medium was entirely replenished. The next experiments were executed 48 h later.

### 2.7 Co‐Immunoprecipitation (Co‐IP)

Introduce fresh frontal cortex tissue or cells into the IP cell lysis buffer (P0013, Beyotime Biotechnology, Shanghai, China). Utilize a high‐speed, low‐temperature tissue grinder to meticulously homogenize the tissue, then continue with the subsequent experimental protocols as per the guidelines for Protein A/G Magnetic Beads (HY‐K0202A—1 mL, MedChemExpress, Shanghai, China).

### 2.8 Dot Blotting

Nitrocellulose membranes were sectioned into 3 × 4 cm pieces. A micropipette was utilized to dispense precise quantities (100 ng, 200 ng, and 500 ng) of the protein sample (USP11) onto the membrane to generate protein spots, in addition to control samples. Membranes containing protein samples were subjected to a drying oven at 37°C for 10 min to ensure complete desiccation and fixation of the samples on the membrane, followed by blocking in 5% skimmed milk at room temperature for 1 h. Subsequent to blocking, the membrane was incubated in a protein solution (CSNK2A1) at room temperature for 3 h. The protein solution was retrieved, and the membrane was rinsed with TBST three times for 10 min each. Anti‐CSNK2A1 (HA722153, Huabio, Hangzhou, China, 1:500) was applied and incubated at room temperature for a minimum of 2 h, followed by three washes of the membrane with TBST solution, each lasting 10 min. An anti‐rabbit secondary antibody (L35009, Signalway Antibody, Nanjing, China, 1:5000) was applied and incubated at room temperature for 1 h, followed by three additional washes in TBST.

### 2.9 Medial Prefrontal Cortex Injection

The procedures for tissue separation and drilling are identical to those previously outlined. The coordinates of the medial prefrontal cortex are defined as (AP, ±1.80 mm; ML, ±0.35 mm; DV, −2.4 mm). 500 μL of the hSyn promoter‐EGFP‐MCS‐SV40 PolyA (AAV‐USP11) was injected into the medial prefrontal cortical region, with 250 μL administered on each side at a rate of 50 μL per minute.

### 2.10 Protein Extraction and Western Blotting

The antibodies are enumerated in Table S2. See Appendix S1 for details.

### 2.11 Immunofluorescence

The antibodies are enumerated in Table S3. See Appendix S1 for details.

### 2.12 Quantitative Real‐Time PCR

The primer pairs utilized in this work are included in Table S4. See Appendix S1 for details.

### 2.13 Mitochondrial Membrane Potential Staining

Separate the mitochondria using the mitochondrial isolation kit (C3606, Beyotime Biotechnology, Shanghai, China). Use the mitochondrial membrane potential assay kit (JC‐1) (C2003S, Beyotime Biotechnology, Shanghai, China) and conduct the measurement of relative mitochondrial membrane potential according to the manufacturer's instructions.

The primary neurons were categorized into three groups: the AAV‐GFP control group, the AAV‐USP11 group, and the AAV‐USP11 + CX4945 group. The TMRE (1X) staining solution (C2001S, Beyotime Biotechnology, Shanghai, China) was incubated at 37°C for 30 min. Following incubation, the cells were examined using a fluorescence microscope.

### 2.14 Transmission Electron Microscopy (TEM)

The frontal lobe cortical tissue was meticulously sectioned into 1 cubic millimeter fragments, then preserved in a 2.5% formaldehyde solution of dopamine, and later subjected to a 1% osmium tetroxide solution treatment. Following dehydration in ethanol, these fragments were soaked overnight in a peroxylene mixture, subsequently embedded in resin and sectioned into 70‐nm slices. The ultrathin sections were treated with 4% uranyl acetate and 0.5% lead citrate on copper grids. The morphology of mitochondria in frontal lobe cortical neurons was examined using a transmission electron microscope (Hitachi, HT7700).

### 2.15 Golgi‐Cox Staining

The prefrontal cortex was dissected into 2–3 mm blocks and rinsed with physiological saline. Tissue blocks were immersed in Golgi staining solution (Servicebio, G1069) for 14 days, with the solution renewed every 3 days. After three rinses with distilled water, tissues were incubated in 80% glacial acetic acid overnight to soften, followed by additional distilled water washes. Samples were then dehydrated in 30% sucrose and sectioned at 100 μm using an oscillating slicer. Sections were mounted onto gelatin‐coated slides and air‐dried in the dark overnight. The next day, slides were treated with concentrated ammonia water for 15 min, rinsed with distilled water for 1 min, incubated in acidic hardening fixative for 15 min, and washed again for 3 min. After air‐drying, slides were sealed with glycerol gelatin. Images were captured using a histological slide scanner (Pannoramic Scanner) with depth‐of‐field expansion mode. For dendritic spine analysis, intact secondary branches with clearly visible spines were selected from the prefrontal cortex. Five neurons per section were quantified. Dendritic length and spine number were measured using Fiji ImageJ software.

### 2.16 Protein Structure and Protein–Protein Docking Predictions

Protein structures of USP11 segments and CK2α were predicted using the AlphaFold Server (https://alphafoldserver.com/). Sequence alignments against experimentally resolved structures were performed, and model quality was assessed using the C‐score to select the most reliable structural predictions. Protein–protein docking between USP11 segments and CK2α was conducted with the HDOCK web server (http://hdock.phys.hust.edu.cn/) to predict putative complex assemblies. Multiple binding conformations were generated and ranked using the ITScorePP scoring function, with docking searches implemented via a fast Fourier transform–based algorithm. Putative interaction interfaces involving USP11 segments and CK2α were further analyzed using the PDBePISA web tool (https://www.ebi.ac.uk/pdbe/pisa/). The top 10 docking models were retained, and computational predictions were integrated with experimental evidence to identify the most plausible interaction model.

### 2.17 Bulk Transcriptomic Analysis

Bulk dorsolateral prefrontal cortex transcriptomic data from GSE54568 were downloaded from GEO. Platform‐specific probe identifiers were mapped to gene symbols using the corresponding annotation, and probe‐level expression was collapsed to gene‐level expression.

### 2.18 Single‐Nuclei RNA‐Seq (snRNA‐Seq) Analysis

#### 2.18.1 Quality Control, Normalization, and Batch Integration

snRNA‐seq data from GSE144136 and GSE213982 were processed using Scanpy (v1.12.0). Cells and genes were filtered to remove low‐quality observations. Highly variable genes were selected for downstream analyses. The data were scaled using the default Scanpy settings before principal component analysis (PCA). PCA was performed and the top 30 principal components were used for k‐nearest neighbor graph construction and UMAP visualization. To mitigate batch effects, Harmony (harmonypy v0.2.0) was applied in the PCA space using the metadata field “Batch” as the batch covariate, and batch‐corrected components were used for downstream visualization and comparisons.

#### 2.18.2 Cell Type Annotation

Major brain cell classes were annotated based on canonical marker genes. Excitatory neurons were identified with SATB2 and SLC17A7; inhibitory neurons with GAD1 and GAD2. Oligodendrocytes were marked by PLP1, MBP, MOG, and MOBP; oligodendrocyte precursor cells by PTPRZ1, PCDH15, OLIG1, OLIG2, and PDGFRA. Astrocytes were distinguished using SLC1A2, ALDH1L1, ALDH1A1, GFAP, GLUL, GJA1, SOX9, AQP4, and NDRG2. Microglia were identified by PTPRC, CSF1R, APBB1IP, P2RY12, CX3CR1, and ITGAM. Endothelial cells were characterized by CLDN5 and VIM. Excitatory neuron subtypes were further localized to cortical layers using layer markers: CUX2, CBLN2, and LINC00507 for layer 2/3; PCP4, PCDH20, and RORB for layer 4; and TLE4, FOXP2, and ETV1 for layer 5/6. TSHZ2 was used as a marker for deep‐layer excitatory neurons. Inhibitory neuron subtypes were annotated using five key markers: CXCL14, LAMP5, PVALB, SST, and VIP.

#### 2.18.3 USP Family Expression Profiling

The USP gene set was defined using standardized gene symbols for ubiquitin specific proteases. USP expression was summarized at the broad cell class level and visualized using clustered heatmaps, with log transformation and gene‐wise scaling applied. Heatmaps were generated separately for the control and MDD groups to facilitate direct comparison of expression patterns between conditions.

### 2.19 Statistical Analyses

Statistical analysis was conducted utilizing GraphPad Prism v9.5.0 software (GraphPad Software, La Jolla, California). All data underwent normality testing via the Shapiro–Wilk test. The unpaired Student's *t*‐test was employed for data analysis between two groups. A one‐way analysis of variance (ANOVA) was utilized for data involving more than two groups, in conjunction with Tukey's multiple comparison test for evaluation. A two‐way analysis of variance was employed for data with two categorical independent variables. The significance level was established at *p* < 0.05. The significance levels were denoted by *p* values: < 0.05 (*), < 0.01 (**), < 0.001 (***).

## 3 Result

### 3.1 Elevated USP11 Expression in the Prefrontal Cortex of Depression Model Mice

The ubiquitination and deubiquitination processes play significant roles in organisms and influence the occurrence and development of diseases by affecting the stability of key protein molecules in biological processes. To explore the deubiquitinases that play a crucial role in depression, we analyzed the human brain transcriptome chip dataset GSE54568. The USP family genes showed significant modular expression heterogeneity, with the most significant changes concentrated in the lower half of the heatmap. The overall sample clustering on the left side was higher, while that on the right side was lower, including genes such as USP48, USP36, USP21, USP20, USP11, USP47, and USP4 (Figure S1). This indicates that the USP‐related pathways may be involved in the molecular heterogeneity of depression and provide clues for subsequent subgroup stratification and mechanism analysis. Subsequently, we analyzed publicly available dLPFC transcriptomic datasets from two independent single‐cell cohorts, GSE144136 and GSE213982, to characterize the expression patterns of USP family genes across different cell types in the human prefrontal cortex in both healthy individuals and patients with major depressive disorder (MDD) (Figure S2). This provides a rationale for selecting specific cell types for manipulation in subsequent experiments.

**FIGURE 1:**
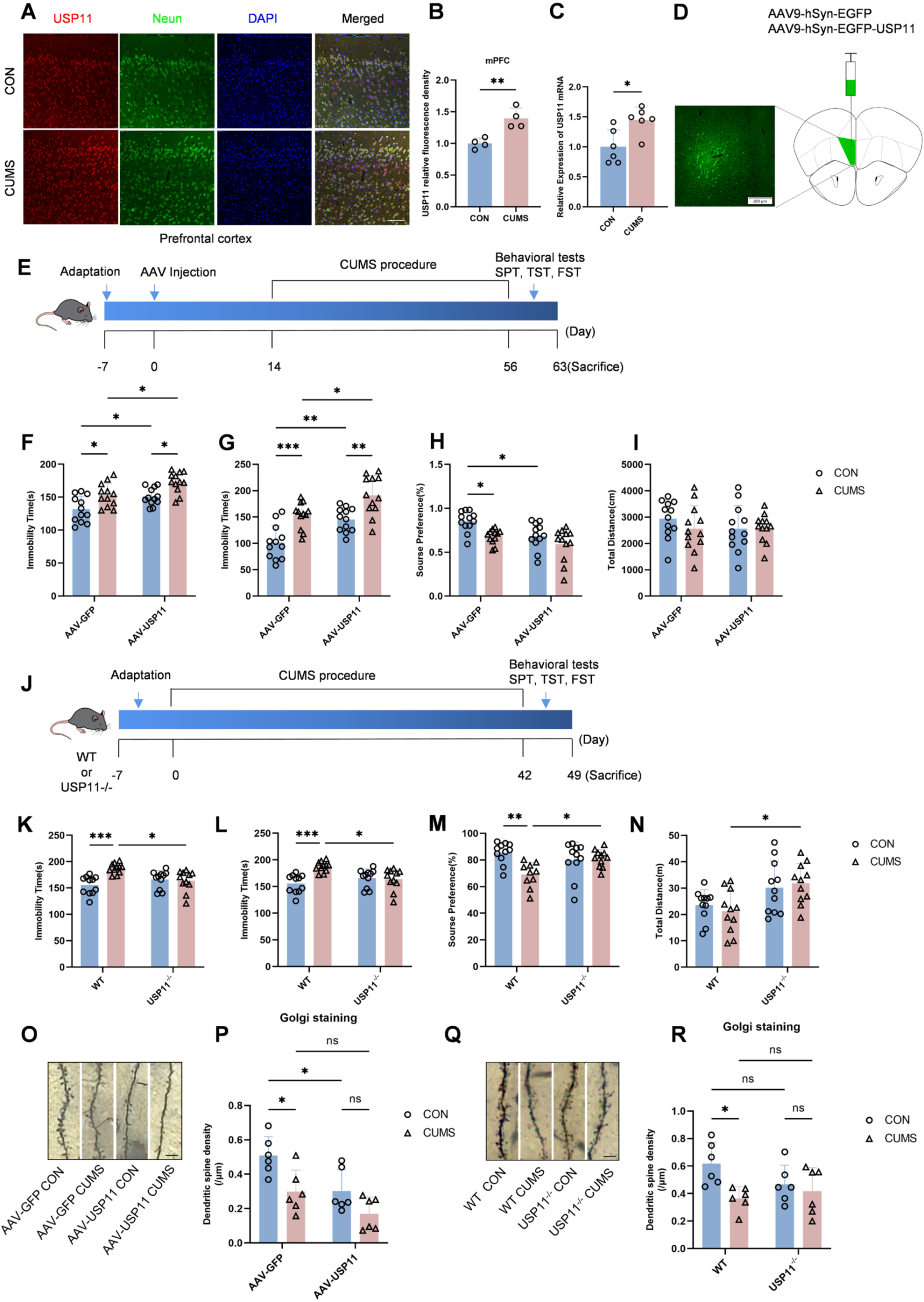
USP11 expression elevated and regulates depressive‐like behaviors in CUMS Mice. (A, B) Immunofluorescence analysis of the expression changes of USP11 in the prefrontal tissue of CUMS‐induced model mice (scale bar = 100 μm, *n* = 3). (C) Changes in the USP11 mRNA content in the prefrontal cortex of CUMS‐induced model mice (*n* = 6). (D) AAV expression in mPFC (scale bar = 200 μm). (E) Flow Chart of Animal Experiments. (F–I) From left to right, there are TST, FST, SPT, OFT results for the four groups, including control and CUMS, each injected with AAV‐GFP or AAV‐USP11 (*n* = 12). (J) Flow Chart of Animal Experiments. (K–N) From left to right, there are TST, FST, SPT, OFT results for the four groups, including control and CUMS groups, each containing two subgroups (wild‐type and USP11 knockout) (*n* = 11). (O, P) Golgi staining images and statistical charts for the four groups, including control and CUMS, each injected with AAV‐GFP or AAV‐USP11 (scale bar = 5 μm, *n* = 6). (Q, R) Golgi staining images and statistical charts for the four groups, including control and CUMS, including control and CUMS groups, each containing two subgroups (wild‐type and USP11 knockout) (scale bar = 5 μm, *n* = 6).

Next, RNA was extracted from the prefrontal cortex tissue of mice after chronic unpredictable mild stress (CUMS) modeling. Real‐time fluorescence quantitative PCR was performed on several highly expressed deubiquitinases in brain tissue. We found that the content of USP11 in the CUMS modeling group was significantly higher than that in the normal control group (Figure 1C, Figure S3). The presence of USP11 was examined by immunofluorescence in multiple depression‐related brain regions, including the prefrontal cortex, hippocampal subregions (CA1, CA3, and DG), amygdala, habenula, and hypothalamus, in both control mice and those exposed to CUMS (Figure S4). Immunofluorescence staining showed that USP11 was significantly increased in the prefrontal cortex of CUMS mice (Figure 1A,B).

The above results indicate that USP11 is overexpressed in the prefrontal cortex of CUMS mice.

### 3.2 USP11 Regulates Depressive‐Like Behaviors in CUMS Mice

To explore whether USP11 can cause depression‐like behavior in mice, we used a stereotaxic apparatus to inject the virus into the bilateral prefrontal cortex of 5‐week‐old C57BL/6J mice, and after 14 days, we constructed a CUMS model. Subsequently, we performed behavioral tests (Figure 1E). Confocal microscopy showed that the control virus carrying green fluorescent protein (GFP) was significantly expressed in the prefrontal cortex tissue sections of mice (Figure 1D). Behavioral tests showed that the sugar preference rate of mice in the AAV‐USP11 group was lower than that of mice in the AAV‐GFP group, indicating that the overexpression of the USP11 gene led to anhedonia in mice (Figure 1H). Compared with the AAV‐GFP group, the AAV‐USP11 group increased the immobility time of mice in the tail suspension test and forced swimming test, indicating that the overexpression of the USP11 gene induced despair behavior in mice (Figure 1F,G). The results of the open field test showed that there was no significant difference in the total moving distance between the AAV‐USP11 group and the AAV‐GFP group, indicating that AAV‐USP11 did not change the motor ability of mice (Figure 1I). Golgi staining showed that, compared with the AAV‐GFP group, the number of dendritic spines in the neurons of the AAV‐USP11 group was significantly reduced (Figure 1O,P). The above results indicate that USP11 overexpression in the prefrontal cortex leads to anhedonia and despair behavior in mice.

To further explore the mechanism of USP11 in the depressive‐like behavior of mice, we constructed USP11 knockout mice (Figure 1J). Six weeks after the establishment of the chronic unpredictable stress model, the WT CUMS group had longer immobility time in the tail suspension test and forced swimming test, and lower sugar preference in the sugar preference test compared with the WT CON group. In contrast, compared with the WT CUMS group, the USP11^−/−^ CUMS group showed shorter immobility time in the tail suspension test and forced swimming test, higher sucrose preference in the sucrose preference test, and greater locomotor activity in the open field test (Figure 1K–N). Golgi staining showed that compared with the WT CON group, the number of dendritic spines in neurons of the WT CUMS group was reduced. Compared with the USP11^−/−^ CON group, there was no significant difference in the number of dendritic spines in neurons of the USP11^−/−^ CUMS group (Figure 1Q,R). The above results indicate that USP11 gene knockout can reverse the depression‐like behavior of CUMS mice.

### 3.3 USP11 Promotes Mitochondrial Dysfunction in the Prefrontal Cortex of CUMS Mice

**FIGURE 2:**
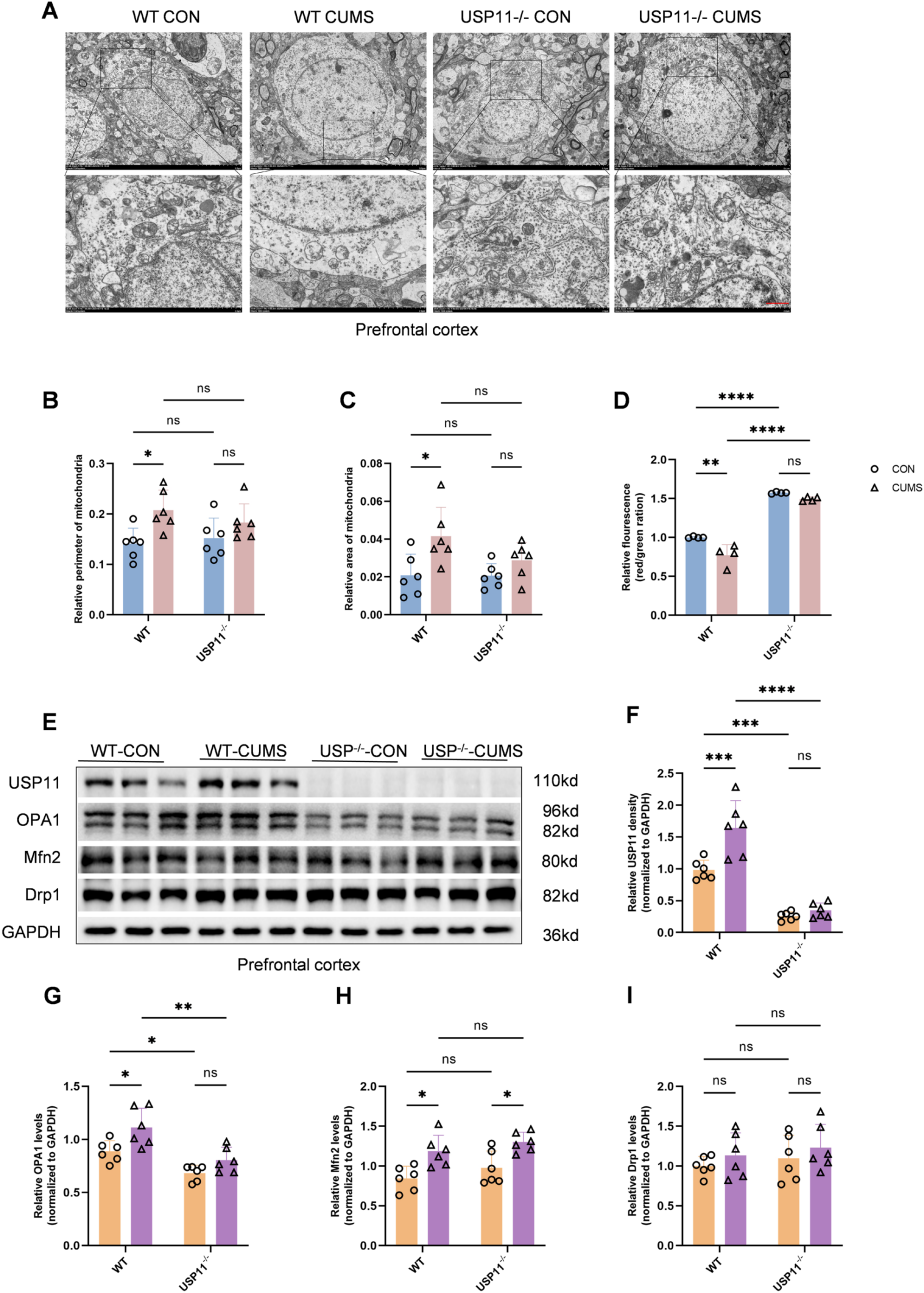
Knockout of USP11 in the prefrontal cortex attenuates the damage to neuronal mitochondria caused by CUMS modeling. (A) Transmission electron microscopy results of mitochondria in PFC neurons of these four groups, including control and CUMS groups, each containing two subgroups (wild‐type and USP11 knockout) (scale bar = 1 μm). (B, C) The statistical results of the mitochondrial perimeter and area in (A) (*n* = 6). (D) Statistical chart of JC‐1 staining results of prefrontal cortex tissue (*n* = 4). (E) Western Blot results of USP11, OPA1, Mfn2, and Drp1 in mouse PFC tissue of above four groups. (F–I) Graphs of the statistical results in (D) (*n* = 6).

To explore the mechanisms by which USP11 induces depressive‐like behaviors and dendritic spine loss, mitochondrial‐related indicators were evaluated in vivo. Mitochondrial morphology was assessed by measuring the perimeter and area of mitochondria. In WT mice, the perimeter and area of mitochondria in the prefrontal cortex of CUMS mice were significantly increased compared with the CON group, the mitochondrial membrane potential level was significantly decreased, and the expression of mitochondrial fusion protein was significantly increased. In USP11^−/−^ mice, there was no significant difference in the perimeter and area of mitochondria in the prefrontal cortex, mitochondrial membrane potential level, and the expression of mitochondrial fission and fusion proteins between the CON group and the CUMS group (Figure 2).

These data indicate that USP11 mediates the mitochondrial fusion and fission process and mitochondrial dysfunction in CUMS mice.

### 3.4 Prediction of CK2α as a USP11 Target

**FIGURE 3:**
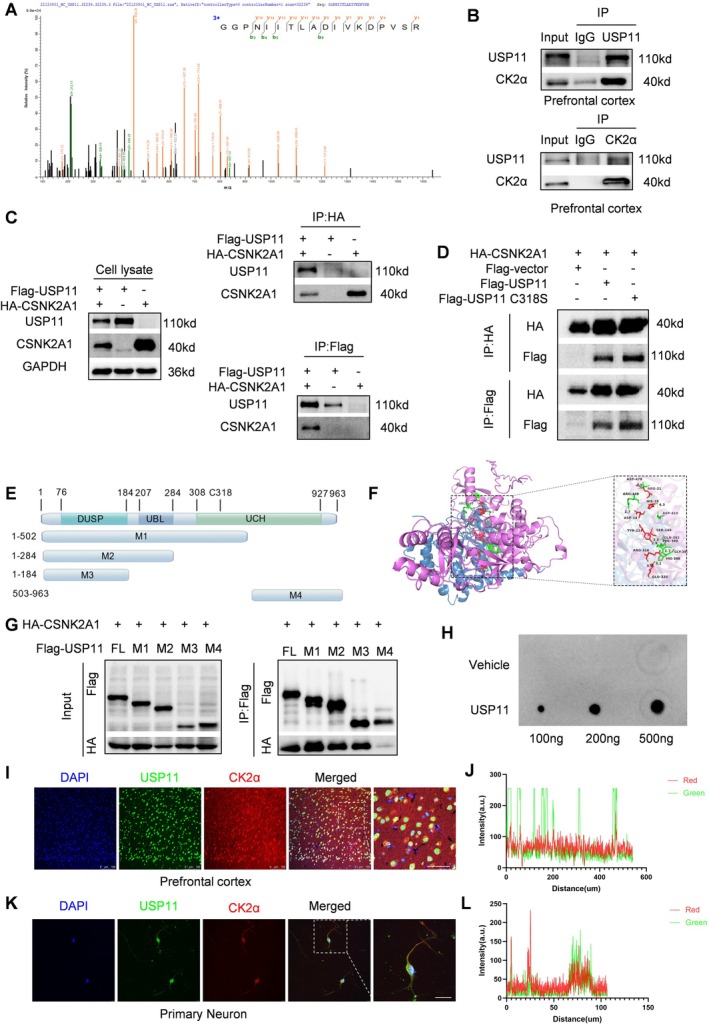
USP11 binds to CK2 both in vivo and in vitro. (A) The secondary mass spectrum obtained after IP using USP11 as the target antibody shows the CK2α‐specific peptide segment GGPNIITLADIVKDPVSR. (B) Endogenous IP results of USP11 in mouse mPFC tissue. (C) IP results in HEK 293T cells after expressing Flag‐USP11 and HA‐CSNK2A1 plasmids. (D) IP results in HEK 293T cells after expressing Flag‐USP11^C318S^ mutant, Flag‐USP11, and HA‐CSNK2A1 plasmids. (E) Schematic diagram of different truncation constructs of USP11. (F) PDBePISA web tool (https://www.ebi.ac.uk/pdbe/pisa/) predicted the interaction between USP11 and CK2α. (G) IP results in HEK 293 T cells after overexpressing Flag‐USP11 fragments and HA‐CSNK2A1. (H) Dot blot analysis of USP11 and CK2α proteins. (I) Immunofluorescence showing the localization of USP11 and CK2α in mouse mPFC tissue (scale bar = 50 μm). (J) The fluorescence intensity distribution maps of USP11 and CK2α in mouse mPFC tissue. (K) Immunofluorescence shows the localization of USP11 and CK2α in primary neurons (scale bar = 25 μm). (L) The fluorescence intensity distribution maps of USP11 and CK2α in primary neurons.

In order to identify the molecules that interact with USP11, we conducted co‐IP on mouse mPFC, followed by mass spectrometry analysis. The IP/MS results indicated that CK2α was identified as a protein that might interact with USP11 (Figure 3A). The interaction between USP11 and CK2α was further confirmed by co‐immunoprecipitation analysis. As shown in the figure, USP11 (but not the control IgG) precipitated CK2α in the mouse prefrontal tissue. Additionally, reverse co‐immunoprecipitation demonstrated that USP11 was significantly precipitated by CK2α in the mouse prefrontal tissue (Figure 3B). In HEK293T cells, we performed reciprocal co‐immunoprecipitation assays using exogenously expressed Flag‐tagged USP11 and HA‐tagged CK2α. As expected, the results demonstrated a physical interaction between USP11 and CK2α (Figure 3C). Next, we transfected a catalytically inactive mutant of Flag‐tagged USP11 into HEK293 cells and found that the inactive mutation of USP11 did not affect its binding to CK2α (Figure 3D). Molecular docking showed the putative binding site of USP11 and CK2α (Figure 3F). To further investigate the region where USP11 binds to CK2α, we studied the full‐length or truncated fragments of USP11 with a Flag tag. Our study demonstrated that the fragment containing the DUSP domain of USP11 could bind to CK2α (Figure 3E,G). Dot blot experiments proved that CK2α could bind to USP11 in vitro (Figure 3H). In conclusion, USP11 interacts with CK2α, and this interaction depends on the M3 domain of USP11. The immunofluorescence analysis of primary mouse neurons showed that CK2α and USP11 were located in the same region within the neurons (Figure 3I,J). Similarly, the immunofluorescence analysis of mouse prefrontal tissue confirmed the same distribution and subcellular localization of CK2α and USP11 (Figure 3K,L).

### 3.5 USP11 Deubiquitinates CK2α

**FIGURE 4:**
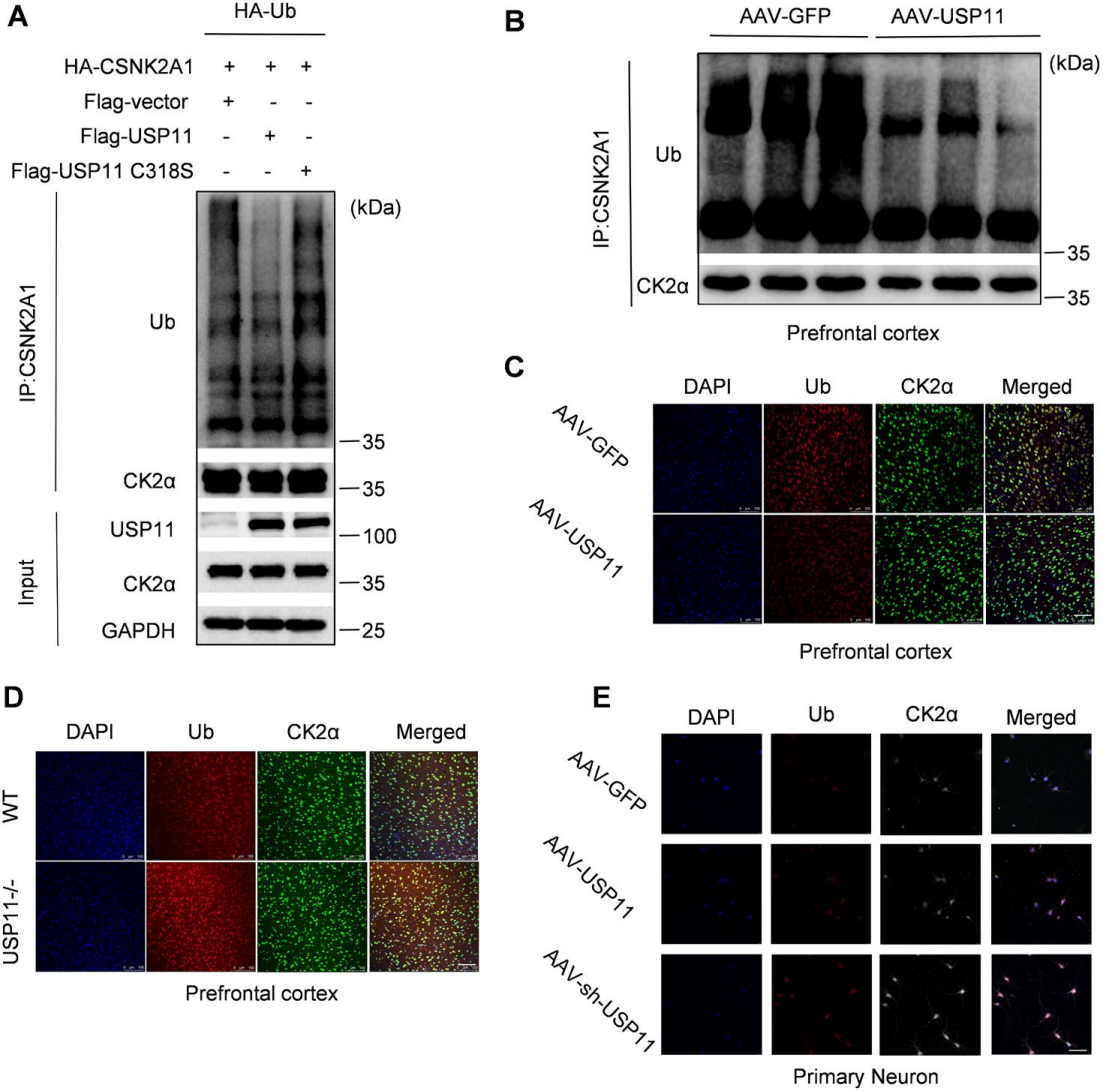
USP11 deubiquitinates CK2α. (A) Western blot showing changes in ubiquitination of CK2α in HEK 293T cells overexpressing HA‐Ub, HA‐CSNK2A1, Flag‐USP11, or Flag‐USP11^C318S^. (B) Western blot analysis revealed the changes in the ubiquitination of CK2α after overexpression of USP11 in mouse mPFC tissue. (C) Immunofluorescence staining showed the changes in ubiquitination of CK2α after overexpression of USP11 in mouse mPFC tissue (scale bar = 100 μm). (D) Immunofluorescence staining showed the changes in ubiquitination of CK2α after the knockout of USP11 in mouse mPFC tissue (scale bar = 100 μm). (E) Immunofluorescence staining revealed the changes in the ubiquitination of CK2α after overexpression or knockdown of USP11 in primary neurons (scale bar = 50 μm).

Next, we investigated how USP11 might regulate CK2α. We transfected HEK293 cells with ubiquitin and CK2α, either in combination with or in the absence of USP11 or the catalytically inactive USP11^C318S^ mutant.^C318S^. After 48 h of plasmid expression, CK2α was pulled down and samples were prepared. Western blot analysis showed that the ubiquitination level of HA‐CK2α in the USP11^C318S^ group was significantly lower than that in the vector group, while the ubiquitination level of HA‐CK2α in the USP11^C318S^ group was significantly higher than that in the USP11 group (Figure 4A), indicating that USP11 can deubiquitinate CK2α. In the mouse prefrontal cortex tissue, we also pulled down CK2α for sample preparation. Western blot analysis showed that the ubiquitination level of CK2α in the overexpressing USP11 group was significantly lower than that in the control group (Figure 4B).

Immunofluorescence staining for ubiquitin (Ub) and CK2α was performed in the mouse prefrontal cortex tissue. The fluorescence intensity of Ub co‐localizing with CK2α in the overexpressing USP11 group was lower than that in the control group (Figure 4C). The fluorescence intensity of Ub co‐localizing with CK2α in the USP11 knockout group was higher than that in the control group (Figure 4D). Immunofluorescence staining for ubiquitin (Ub) and CK2α was performed in neurons. The fluorescence intensity of Ub co‐localizing with CK2α in the overexpressing USP11 group was lower than that in the control group, while the fluorescence intensity of Ub co‐localizing with CK2α in the knockdown USP11 group was higher than that in the control group (Figure 4E).

### 3.6 USP11 Stabilizes CK2α

**FIGURE 5:**
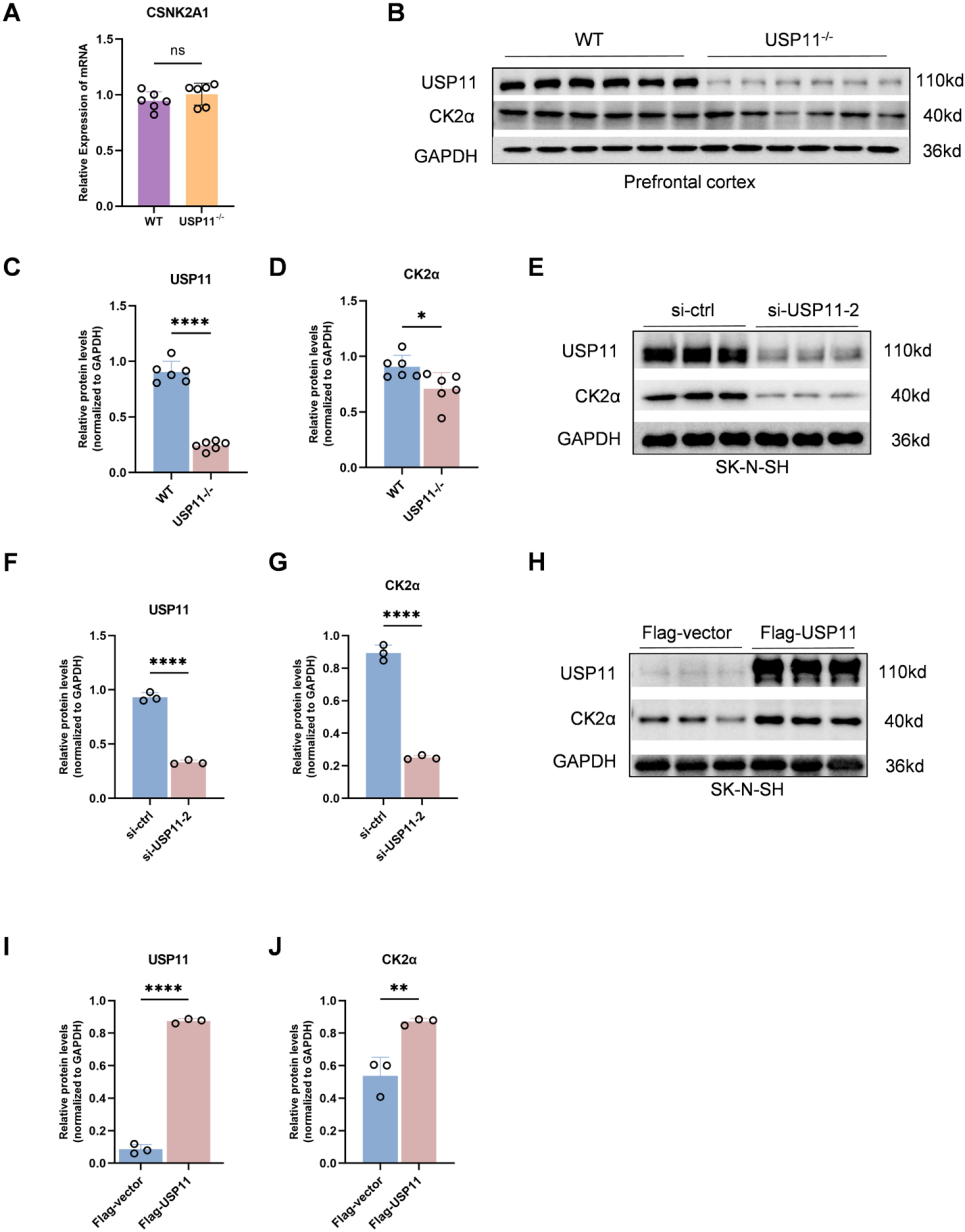
USP11 stabilizes CK2α. (A) qRT‐PCR analysis of CSNK2A1 in USP11 knockout mouse mPFC tissue (*n* = 6). (B) Western Blot results of USP11 and CK2α in USP11 knockout mouse mPFC tissue. (C, D) Graphs of the statistical results in (B) (*n* = 6). (E) Western Blot results of USP11 and CK2α in SK‐N‐SH cells. (F, G) Graphs of the statistical results in (E) (*n* = 3). (H) Western Blot results of USP11 and CK2α in SK‐N‐SH cells. (I, J) Graphs of the statistical results in (H) (*n* = 3).

The qPCR results showed that there was no statistically significant difference in the CSNK2A1 mRNA content in the prefrontal tissue of USP11 knockout mice compared to wild‐type mice (Figure 5A). The Western Blot results indicated that the CK2α protein content in the prefrontal tissue of USP11 knockout mice was lower than that of wild‐type mice (Figure 5B–D). These results indicate that the level of CK2α protein is regulated by post‐translational modifications rather than transcription.

The impact of siRNA‐mediated USP11 knockdown on CK2α expression in SK‐N‐SH cells was investigated. Compared with the siRNA‐Ctrl group, the protein expression of CK2α in the siRNA‐USP11 group was significantly decreased (Figure 5E–5G). In contrast, the overexpression of USP11 stabilized the expression of CK2α (Figure 5H–J).

### 3.7 USP11 Regulates Mitochondrial Function via CK2α in Primary Mouse Neuron

**FIGURE 6:**
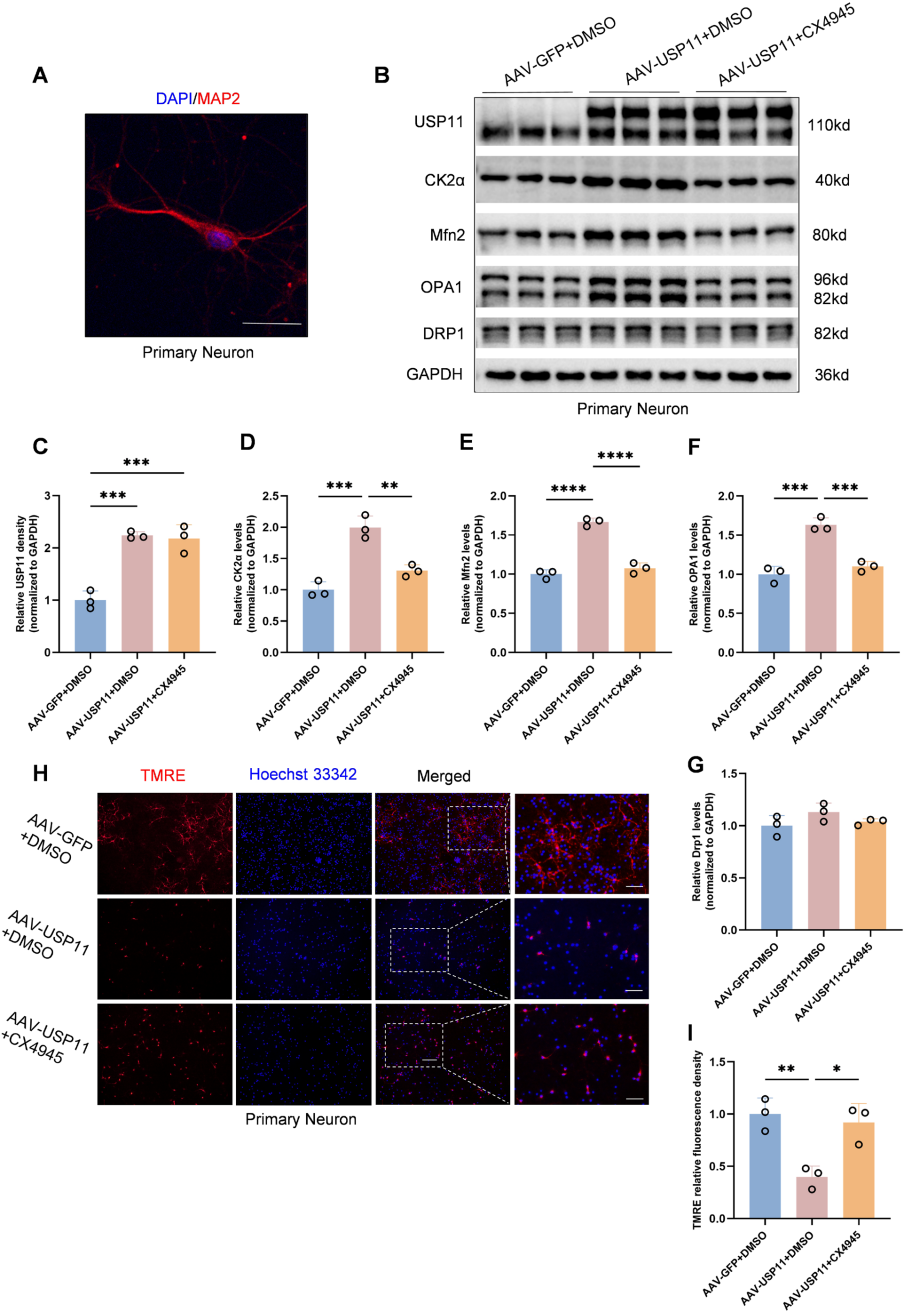
USP11 regulates mitochondrial function via CK2α in primary mouse neuron. (A) Immunofluorescence staining of MAP2 for the extracted primary neurons (scale bar = 25 μm). (B) After overexpressing USP11 in primary neurons, solvent DMSO or CK2 inhibitor CX4945 was added respectively. Western blotting results of USP11, CK2α, OPA1, Mfn2, and Drp1 in the three groups are shown in the figure. (C–G) Graphs of the statistical results in (B) (*n* = 3). (H, I) The results of primary neurons TMRE staining for mitochondrial membrane potential in the three groups (scale bar = 50 μm, *n* = 3).

To confirm that USP11 regulates neuronal mitochondrial function through CK2α, we isolated primary cortical neurons from mice (Figure 6A). Western blotting indicated that overexpression of USP11 increased CK2α protein levels and promoted mitochondrial fusion in primary neurons, while CX4945 (CK2 inhibitor) reversed this effect (Figure 6B–G). TMRE staining showed that overexpression of USP11 decreased mitochondrial membrane potential, and CX4945 reversed this change (Figure 6H,I). These findings are consistent with the in vivo results and suggest that USP11 regulates neuronal mitochondrial function through CK2α.

## 4 Discussion

This study explored the potential mechanisms by which USP11 promotes the occurrence and development of depression and its role in mitochondrial fusion and fission. The protein ubiquitination process controls many intracellular processes, including cell cycle progression, transcriptional activation, and signal transduction. Previous studies have shown that USP7, USP8, and USP25 play a role in depression [57]. However, the mechanism of USP11 in depression has not been reported. It is extremely challenging to directly study the changes in USP11 levels in the brain tissue of patients with depression. Therefore, we constructed the CUMS mouse model and detected the USP11 mRNA level and immunofluorescence intensity in the prefrontal cortex. These results preliminarily suggested that USP11 played a key role in depression.

Moreover, USP11 has also been studied in the nervous system. Yan Yan et al. found that USP11 can deubiquitinate and promote the acetylation of lysine residues 281 and 274 of tau protein, which helps to promote the abnormal aggregation of tau protein in the development of Alzheimer's disease [28]. Xiuqing Zhang et al. discovered that USP11 significantly inhibits the transcription of the KLF2‐NF‐κB signaling pathway by stabilizing p53, thereby promoting the release of pro‐inflammatory factors and neurofunctional disorders caused by cerebral hemorrhage. Targeting USP11/P53/KLF2/NF‐κB may be a new anti‐inflammatory method for treating cerebral hemorrhage [58]. Additionally, ShangYin Chiang et al. discovered that USP11 controls cortical neurogenesis and neuronal migration by stabilizing Sox11 [22]. In this study, we demonstrated that USP11 induced depression‐like behavior and mitochondrial dysfunction in mice via mouse behavioral experiments and mitochondrial function‐related detection. USP11 is a specific cysteine protease that can cleave off ubiquitin from the protein substrate bound to it. The catalytic and binding activities of USP11 involve two key domains, namely the DUSP and USP domains. The cysteine 318 position is the core amino acid involved in the catalytic activity, and mutations or deletions at this site can lead to the loss of deubiquitination function [20, 21, 23]. This study shows that the USP11 binding site for CK2α is amino acids 1–502, including its USP domain. The cysteine 318 mutation of USP11 weakens the deubiquitination of CK2α. Therefore, small molecule compounds targeting the USP domain of the USP11 protein may represent the main direction for future clinical treatment of depression.

CK2 is a constitutively active serine/threonine protein kinase that participates in regulating multiple signaling pathways and is associated with numerous human diseases. The activity of CK2 is regulated by post‐translational modifications and protein–protein interactions. Previous studies have shown that CK2 can regulate its activity through autophosphorylation, including phosphorylation at the Y182 site of CK2α and the Y183 site of CK2α’ [29]. Additionally, CK2 can be activated by phosphorylation from other enzymes. For instance, after activation by the mitogen‐activated protein kinase (MAPK) pathway, extracellular signal‐regulated kinase 2 (ERK2) phosphorylates CK2α at the T360 and S362 sites, thereby enhancing its phosphorylation of α‐connexin [59]. Research also indicates that CK2α is phosphorylated at the Y255 site by Src family kinases Lyn and C‐Fgr, resulting in a threefold increase in its activity [60]. Similarly, CK2α is phosphorylated at the Y182 and Y188 sites by the Src‐related kinase SRMS [61]. However, phosphorylation at the Y188 site leads to a significant increase in CK2α activity. Moreover, acetylation at the lysine 102 site also enhances its kinase activity [62]. Post‐translational modifications have also been shown to reduce CK2 activity. For example, the S347 site in CK2α (adjacent to the CDK1 phosphorylation site T344) is O‐GlcNAc glycosylated. This glycosylation inhibits CDK1‐mediated phosphorylation of CK2 and increases its susceptibility to proteasomal degradation [63]. However, there are relatively few studies on the ubiquitination modification of CK2. Compared with previous studies, our research indicates that the de‐ubiquitination modification of CK2α by USP11 enhances its stability, explaining the reason for the increased content of CK2α in depression. At the same time, our research shows that CK2α promotes the occurrence and development of depression by enhancing the expression of the mitochondrial fusion protein OPA1, providing new evidence for the role of CK2α in the occurrence and development of depression.

This study has several limitations. First, while we demonstrate that USP11 deubiquitinates and stabilizes CK2α, the selective CK2α inhibitor CX4945 did not block this deubiquitination, suggesting that the USP11‐CK2α interaction itself may represent a more direct therapeutic target. Second, the use of whole body USP11 knockout mice precludes definitive conclusions about the PFC specific role of USP11, although our complementary PFC restricted overexpression experiments partially address this concern. Third, the precise PFC subregions and neuronal subtypes through which USP11 exerts its effects remain to be determined. Finally, although our in vitro data establish that CK2α contributes to USP11 induced mitochondrial dysfunction, evidence linking CK2α to USP11 mediated stress susceptibility is still lacking. Future studies employing PFC‐specific CK2α knockdown or inhibition in USP11 overexpressing mice will be necessary to establish causality in vivo.

## 5 Conclusion

In conclusion, our research demonstrates that USP11 directly binds to and deubiquitinates CK2, affecting mitochondrial function in the mPFC and leading to depressive‐like behaviors in mice. This discovery reveals a new mechanism underlying the pathogenesis of depression and provides a more precise target for the treatment of patients with depression.

## Funding

This work was supported by grants from the National Natural Science Foundation of China (grant number: U21A20364 and 82501827) and China Postdoctoral Science Foundation under Grant Number 2025M782239. This work has not received funding/assistance from any commercial organizations. The funding sources had no roles in the design of this study and will not have any roles during the execution, analyses, interpretation of the data, or decision to submit results.

## Ethics Statement

The animal study protocol was approved by the Institutional Review Board of Renmin Hospital of Wuhan University (protocol code WDRM20240103A and date of approval is 01/17/2024).

## Conflicts of Interest

The authors declare no conflicts of interest.

## Supporting information


**Figure S1:** The heatmap shows the bulk dlPFC transcriptomic dataset GSE54568. The heatmap depicts log transformed, gene wise scaled expression of USP family genes across individuals, with unsupervised hierarchical clustering applied to both genes and samples. The top annotation denotes diagnostic status (control versus MDD), revealing coordinated gene blocks that define discrete sample level expression states and indicating a reproducible modular organization of USP transcription in bulk tissue.
**Figure S2:** The two heatmap summarize USP family expression in the snRNA seq dataset GSE144136 and GSE213982, aggregated at the broad cell class level (Ast, End, ExN, InN, Mic, Mix, OPC, and Oli) for control and MDD groups, respectively. Values are shown after log transformation and gene wise scaling to facilitate comparison of relative expression patterns across cell classes and gene modules. Together, these two heatmap demonstrate pronounced cell class specificity of USP programs and a case control associated shift in the relative module patterns across major cell classe.
**Figure S3:** Statistical graph of USPs mRNA content in the prefrontal cortex of CUMS mice.
**Figure S4:** Statistical graph of USPs mRNA content in the prefrontal cortex of CUMS mice.
**Table S1:** The specific schedule for CUMS.
**Table S2:** Details of Antibody and dilution rate for western blotting.
**Table S3:** Details of Antibody and dilution rate for immunofluorescent staining.
**Table S4:** Genes primers used for real time PCR analyses.

## Data Availability

The data supporting the findings of the study are accessible from the corresponding author upon a reasonable request.
