# Supplementary material for: X‐Linked USP11 Drives Depression‐Like Behaviors by Stabilizing CK2α and Disrupting Mitochondrial Function
Source: CNS Neurosci Ther. 2026 Jun 2;32(6):e70934. doi: 10.1002/cns.70934 (PMC13239159; doi:10.1002/cns.70934)
Supplement: Supplementary file 1 — Figure S1: The heatmap shows the bulk dlPFC transcriptomic dataset GSE54568. The heatmap depicts log transformed, gene wise scaled expression of USP family genes across individuals, with unsupervised hierarchical clustering applied to both genes and samples. The top annotation denotes diagnostic status (control versus MDD), revealing coordinated gene blocks that define discrete sample level expression states and indicating a reproducible modular organization of USP transcription in bulk tissue. Figure S2: The two heatmap summarize USP family expression in the snRNA seq dataset GSE144136 and GSE213982, aggregated at the broad cell class level (Ast, End, ExN, InN, Mic, Mix, OPC, and Oli) for control and MDD groups, respectively. Values are shown after log transformation and gene wise scaling to facilitate comparison of relative expression patterns across cell classes and gene modules. Together, these two heatmap demonstrate pronounced cell class specificity of USP programs and a case control associated shift in the relative module patterns across major cell classe. Figure S3: Statistical graph of USPs mRNA content in the prefrontal cortex of CUMS mice. Figure S4: Statistical graph of USPs mRNA content in the prefrontal cortex of CUMS mice. Table S1: The specific schedule for CUMS. Table S2: Details of Antibody and dilution rate for western blotting. Table S3: Details of Antibody and dilution rate for immunofluorescent staining. Table S4: Genes primers used for real time PCR analyses. [file CNS-32-e70934-s001.docx]

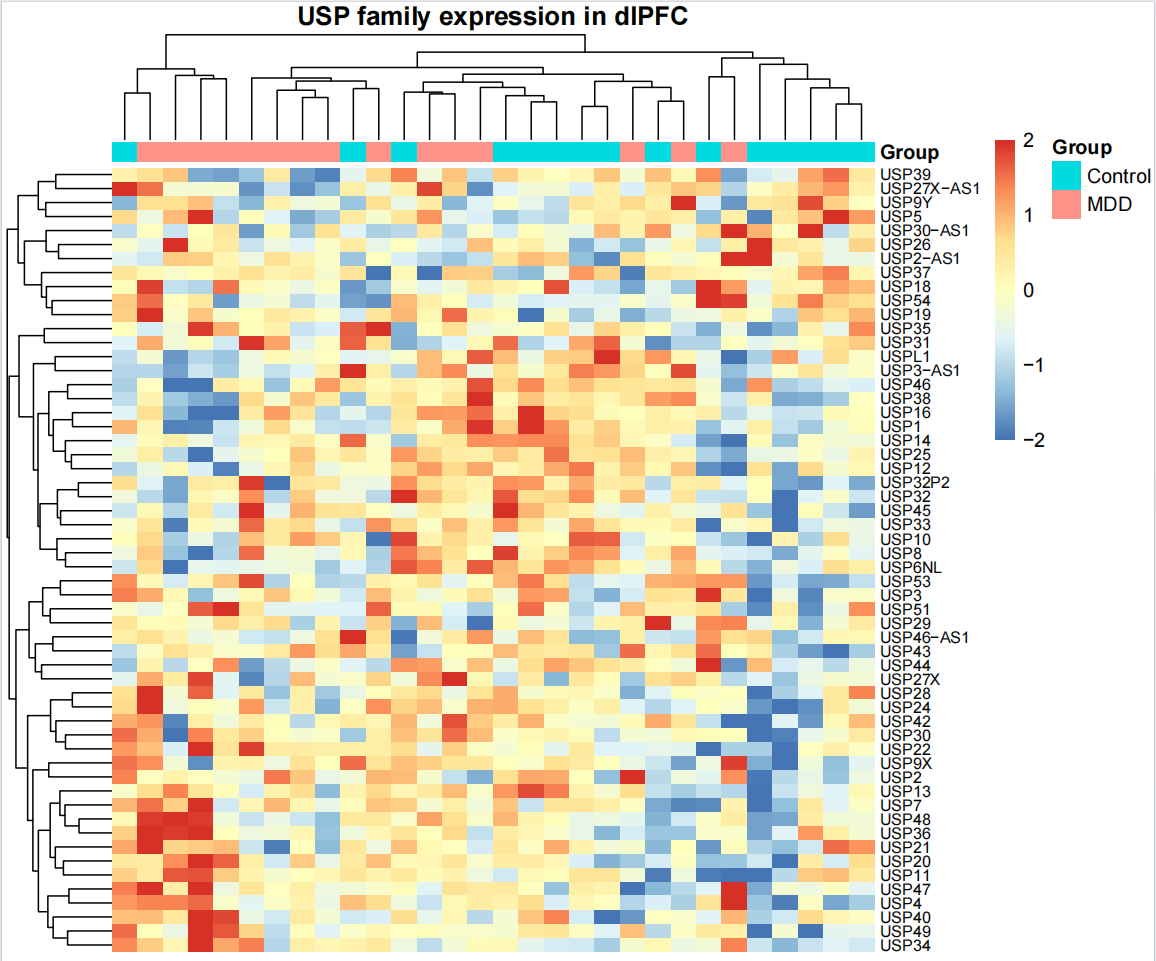


**Figure S1**. The heatmap shows the bulk dlPFC transcriptomic dataset GSE54568. The heatmap depicts log transformed, gene wise scaled expression of USP family genes across individuals, with unsupervised hierarchical clustering applied to both genes and samples. The top annotation denotes diagnostic status (control versus MDD), revealing coordinated gene blocks that define discrete sample level expression states and indicating a reproducible modular organization of USP transcription in bulk tissue.

A
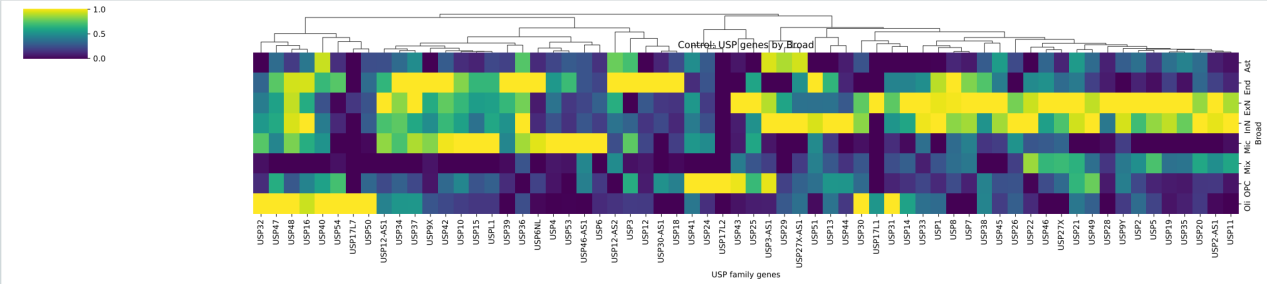


B


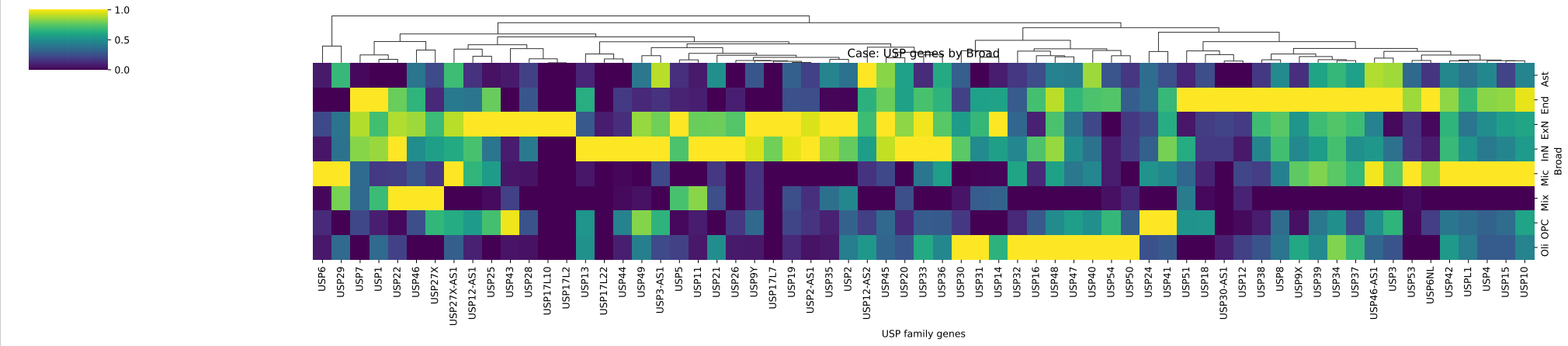


**Figure S2**. The two heatmap summarize USP family expression in the snRNA seq dataset GSE144136 and GSE213982, aggregated at the broad cell class level (Ast, End, ExN, InN, Mic, Mix, OPC, Oli) for control and MDD groups, respectively. Values are shown after log transformation and gene wise scaling to facilitate comparison of relative expression patterns across cell classes and gene modules. Together, these two heatmap demonstrate pronounced cell class specificity of USP programs and a case control associated shift in the relative module patterns across major cell classe.


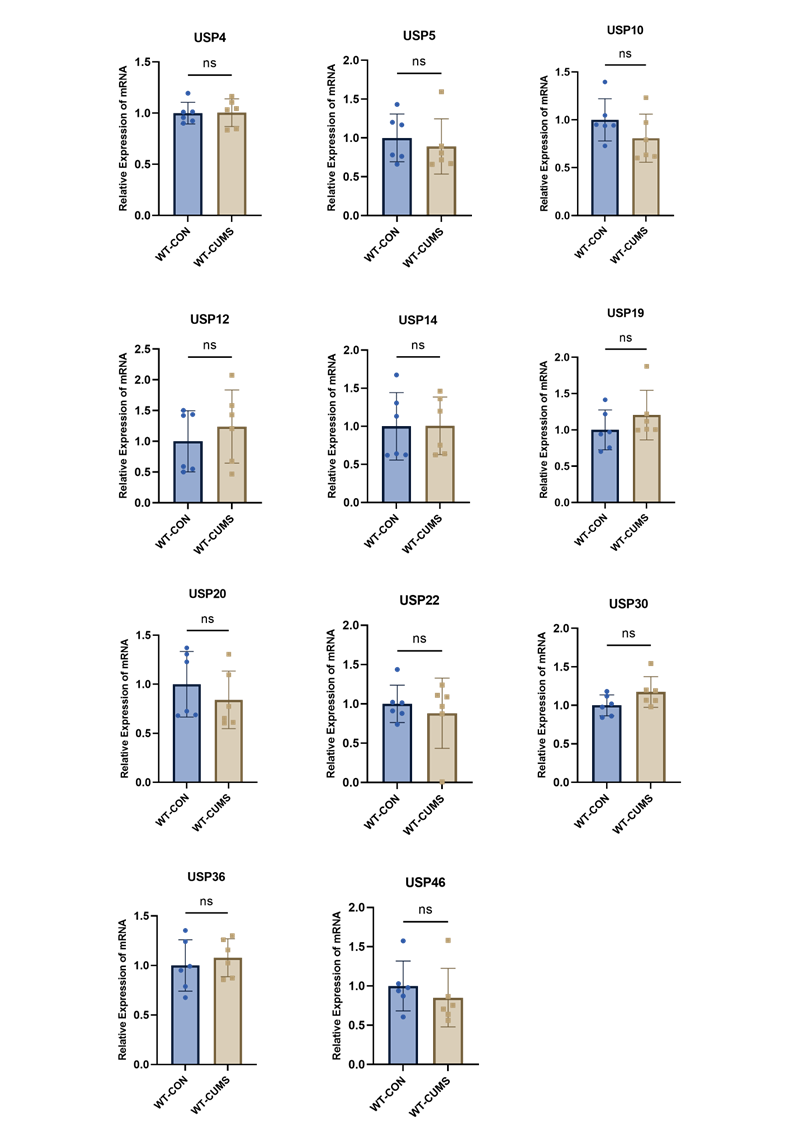


**Figure S3**. Statistical graph of USPs mRNA content in the prefrontal cortex of CUMS mice.


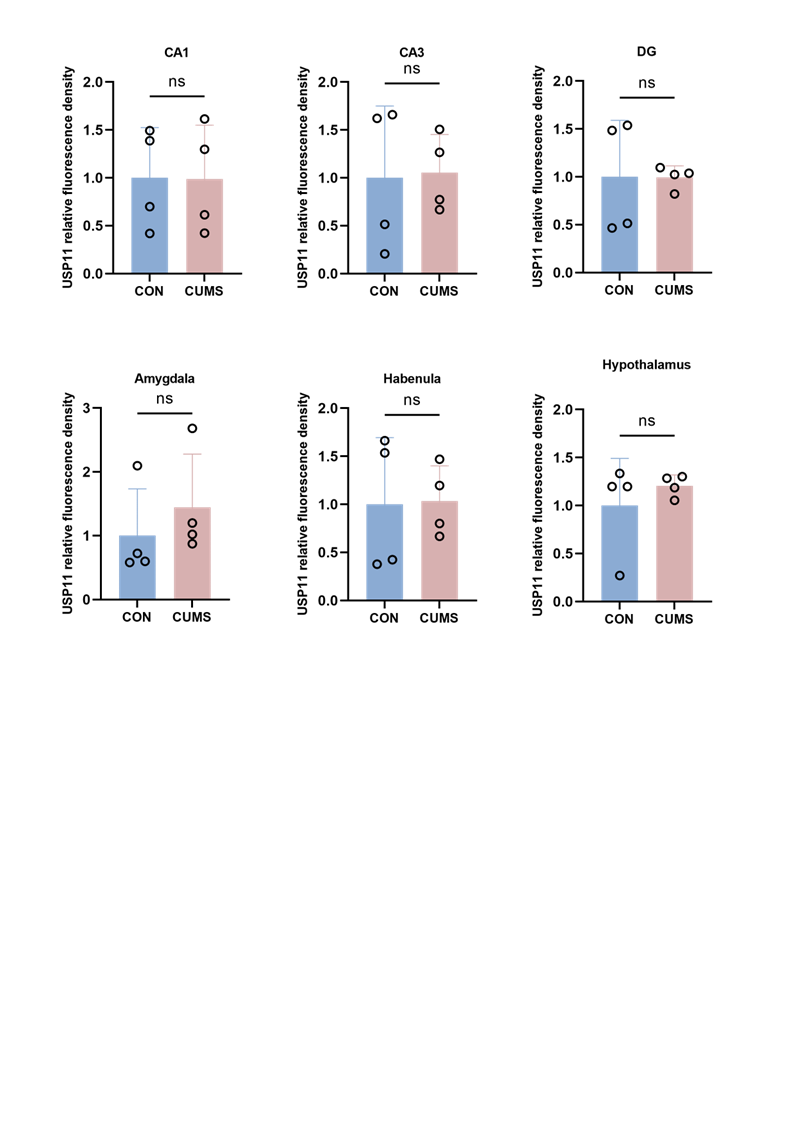


**Figure S4**. Statistical graph of USPs mRNA content in the prefrontal cortex of CUMS mice.

**Table S1**. The specific schedule for CUMS

| Week | Day | Stressor 1 | Stressor 2 |
| --- | --- | --- | --- |
| 1 | Monday | 24-hour cage tilting at 45° | 6-hour physical restraint |
|  | Tuesday | 24-hour light/dark cycle reversal | 30-min cage shaking at 120 rpm |
|  | Wednesday | 24-hour solid cage | 5-min swimming in 4°C water |
|  | Thursday | 24-hour water deprivation | 10-min tail pinching |
|  | Friday | 24-hour damp bedding | 6-hour physical restraint |
|  | Saturday | 24-hour light/dark cycle reversal | 24-hour cage tilting at 45° |
|  | Sunday | 24-hour food deprivation | 10-min tail pinching |
| 2 | Monday | 24-hour solid cage | 24-hour water deprivation |
|  | Tuesday | 24-hour cage tilting at 45° | 6-hour physical restraint |
|  | Wednesday | 24-hour light/dark cycle reversal | 30-min cage shaking at 120 rpm |
|  | Thursday | 24-hour food deprivation | 10-min tail pinching |
|  | Friday | 24-hour damp bedding | 5-min swimming in 4°C water |
|  | Saturday | 24-hour water deprivation | 6-hour physical restraint |
|  | Sunday | 24-hour food deprivation | 24-hour cage tilting at 45° |
| 3 | Monday | 24-hour solid cage | 10-min tail pinching |
|  | Tuesday | 24-hour light/dark cycle reversal | 30-min cage shaking at 120 rpm |
|  | Wednesday | 24-hour damp bedding | 6-hour physical restraint |
|  | Thursday | 24-hour food deprivation | 5-min swimming in 4°C water |
|  | Friday | 24-hour cage tilting at 45° | 10-min tail pinching |
|  | Saturday | 24-hour water deprivation | 24-hour light/dark cycle reversal |
|  | Sunday | 24-hour damp bedding | 6-hour physical restraint |
| 4 | Monday | 24-hour solid cage | 30-min cage shaking at 120 rpm |
|  | Tuesday | 24-hour cage tilting at 45° | 5-min swimming in 4°C water |
|  | Wednesday | 24-hour water deprivation | 10-min tail pinching |
|  | Thursday | 24-hour food deprivation | 6-hour physical restraint |
|  | Friday | 24-hour light/dark cycle reversal | 30-min cage shaking at 120 rpm |
|  | Saturday | 24-hour damp bedding | 10-min tail pinching |
|  | Sunday | 24-hour cage tilting at 45° | 24-hour water deprivation |
| 5 | Monday | 24-hour food deprivation | 6-hour physical restraint |
|  | Tuesday | 24-hour solid cage | 5-min swimming in 4°C water |
|  | Wednesday | 24-hour water deprivation | 30-min cage shaking at 120 rpm |
|  | Thursday | 24-hour damp bedding | 10-min tail pinching |
|  | Friday | 24-hour light/dark cycle reversal | 24-hour solid cage |
|  | Saturday | 24-hour cage tilting at 45° | 5-min swimming in 4°C water |
|  | Sunday | 24-hour water deprivation | 6-hour physical restraint |
| 6 | Monday | 24-hour food deprivation | 24-hour light/dark cycle reversal |
|  | Tuesday | 24-hour damp bedding | 10-min tail pinching |
|  | Wednesday | 24-hour water deprivation | 30-min cage shaking at 120 rpm |
|  | Thursday | 24-hour solid cage | 5-min swimming in 4°C water |
|  | Friday | 24-hour cage tilting at 45° | 6-hour physical restraint |
|  | Saturday | 24-hour light/dark cycle reversal | 24-hour damp bedding |
|  | Sunday | 24-hour food deprivation | 10-min tail pinching |

**Table S2**. Details of Antibody and dilution rate for western blotting

| Antibody | Manufacturer | Product numbers | Dilution Rate |
| --- | --- | --- | --- |
| Rabbit anti-USP11 | ABclonal | A19562 | 1:1000 |
| Rabbit anti-CSNK2A1 | HUABIO | HA722153 | 1:5000 |
| Rabbit anti-OPA1 | HUABIO | ET1705-9 | 1:1000 |
| Rabbit anti-Mfn2 | HUABIO | HA720073 | 1:1000 |
| Rabbit anti-Drp1 | HUABIO | HA500487 | 1:1000 |
| Rabbit anti-Ubiquitin | ABclonal | A19686 | 1:1000 |
| Rabbit anti-HA-Tag | ABclonal | AE105 | 1:5000 |
| Rabbit anti-DDDDK-Tag | ABclonal | AE092 | 1:5000 |
| GAPDH-HRP | HUABIO | ET1702-66 | 1:40000 |
| Goat Anti-Rabbit IgG (H&L) HRP | Signalway | L35009 | 1:5000 |
| Goat Anti-Mouse IgG (H&L) HRP | Signalway | L35007 | 1:5000 |

**Table S3**. Details of Antibody and dilution rate for immunofluorescent staining

| Antibody | Manufacturer | Product numbers | Dilution Rate |
| --- | --- | --- | --- |
| Rabbit anti-USP11 | ABclonal | A19562 | 1:200 |
| Mouse anti-CSNK2A1 | Santa Cruz Biotechnology | sc-365762 | 1:500 |
| Rabbit anti-Ubiquitin | ABclonal | A23483 | 1:100 |
| Goat anti-mouse IgG-Alexa Flour 488 | Thermo Scientific | A-11001 | 1:500 |
| Goat anti-rabbit IgG-Alexa Flour 594 | Thermo Scientific | A-11012 | 1:500 |

**Table S4**. Genes primers used for real time PCR analyses

| Gene | Primer sequences |
| --- | --- |
| Gapdh | Forward: 5’-GGGGTCGTTGATGGCAACA-3’  Reverse: 5’-AGGTCGGTGTGAACGGATTTG-3’ |
| CSNK2A1 | Forward: 5’-GAGCCGTGTGTCTCCTCCTC-3’  Reverse: 5’-GACCCTTTTTCTTCACACCGC-3’ |
| USP11 | Forward: 5’-GCTGGCGAAAGCTGATAACAC-3’  Reverse: 5’-TCCTCTCCTGGCAGGCTAAT-3’ |

**Methods Details**

**2.10 Protein extraction and western blottingting**

The cells or tissues of the medial prefrontal cortex from mice were lysed in RIPA buffer (G2002, Servicebio, Wuhan, China) containing protease and phosphatase inhibitors. The lysates underwent ultrasonication and were centrifuged at 13,200 rpm for 20 minutes at 4°C to obtain the supernatant. The protein concentration was quantified with the BCA technique. During electrophoresis, a minimum of 20 micrograms of protein was introduced per well. The protein samples were transferred to PVDF membranes (MilliporeSigma, Burlington, Massachusetts). The samples were treated with a 5% BSA solution, and the membranes were incubated with the primary antibody solution overnight at 4°C. The membranes were subjected to five washes with TBST (5 minutes each) the next day, thereafter incubated with the secondary antibody at room temperature for 1 hour, and then washed three additional times with TBST. The images were produced via a chemiluminescence imaging apparatus. The images were examined utilizing ImageJ.

**2.11 Immunofluorescence**

Paraffin portions were immersed in xylene solution thrice for 25 minutes each. Subsequently, parts were rehydrated in anhydrous ethanol, 95% ethanol, 85% ethanol, 75% ethanol, and ultrapure water, with each step lasting 5 minutes. For antigen retrieval, slices were immersed in 0.1 M sodium citrate solution at 94°C for 20 minutes, thereafter allowing them to cool to room temperature naturally. Brain or cell slices were fixed with 4% PFA three times using phosphate-buffered saline (PBS) and subsequently blocked with 3% BSA containing 3% Triton-X 100 at room temperature for one hour.

Sections were incubated with the primary antibody at 4°C for 24 hours, followed by three washes with PBS at room temperature, each lasting 5 minutes. Sections were treated with fluorescent secondary antibodies for two hours at ambient temperature. Following three washes with PBS, DAPI staining solution (Beyotime) was applied at room temperature in the dark for 10 minutes. Sections were subsequently washed in the dark three times following gentle agitation, air-dried, sealed with an anti-fluorescence quenching mounting solution (Beyotime), and photographed using a fluorescence microscope (Olympus, BX51).

**2.12 Quantitative real-time PCR**

TRIzol reagent (G3013, Servicebio, Wuhan, China) was utilized for the extraction of total RNA from tissues or cells, followed by phase separation with the addition of chloroform. Following centrifugation, the supernatant was transferred to a fresh tube, isopropanol was added, and RNA was precipitated, subsequently washed with 75% ethanol and dried. RNA was subsequently solubilized in DEPC water, and the RevertAid First Strand cDNA Synthesis Kit (K1621, Thermo Scientific, Waltham, MA, USA) was employed to reverse transcribe RNA into cDNA. Ultimately, Taq Pro Universal SYBR qPCR Master Mix (Q712-02, Vazyme, Nanjing, China) was employed for PCR amplification. GAPDH was chosen as an internal standard for normalization, and the 2-ΔΔCT technique was employed to ascertain relative gene expression levels
